# Description of a rat model of polymicrobial abdominal sepsis mimicking human colon perforation

**DOI:** 10.1186/s13104-020-05438-y

**Published:** 2021-01-07

**Authors:** Julia M. Utiger, Michael Glas, Anja Levis, Josef Prazak, Matthias Haenggi

**Affiliations:** 1 Department of Intensive Care Medicine, Bern University Hospital, Inselspital, University of Bern, Bern, Switzerland; 2 Department of Anaesthesiology and Pain Medicine, Bern University Hospital, Inselspital, University of Bern, Bern, Switzerland

**Keywords:** Rodent, Hemodynamic monitoring, Cecum, Colon, Ascending, Punctures, Peritonitis, Sepsis

## Abstract

**Objective:**

Standard rodent sepsis models as cecal ligation and puncture models (CLP) or cecal ligation and incision models (CLI) are frequently not suited experiments, mainly because they lack surgical repair, and they are difficult to control for severity. The colon ascendens stent peritonitis model (CASP) overcomes some of these limitations.

**Result:**

Here we present our modification of the rodent CASP model, where severity of sepsis can be controlled by timing of surgical repair and treatment, and by diameter of the stent. Further, basic hemodynamic monitoring (blood pressure and heart rate) and frequent blood sampling can be achieved, which might guide further treatment.

## Introduction

Sepsis is a life-threatening organ dysfunction due to a dysregulated host response to infection [1] and is associated with high mortality and unfavorable outcome [2, 3]. Neither sepsis burden nor mortality has changed in the past decade [3]. Given the higher risk of acquiring sepsis and of adverse outcome in the elderly [4], with the aging population in many countries sepsis will remain and even will rise as one of the most important health care threats.

Since sepsis is a complex syndrome defined as organ dysfunction associated with dysregulated host response to infection [1, 4], preclinical research has to rely on animal models to explore the complex interaction of infectious agents and host factors. Commonly used research models are endotoxin/toxin administration, exogenously administered pathogenic bacteria, and host-barrier disruption models, where the animals’ protective endogenous host barrier is altered. For practical and financial reasons, rodent models dominate over other species. The far most frequent models within this category are models where the intestinum is disrupted, and the intestinal content spills into the normally sterile peritoneal cavity and thus induces a polymicrobial sepsis. Cecal ligation and puncture (CLP models) and cecal ligation and incision (CLI models) are regarded as gold standard models because they resemble initiation and progression with characteristics of human sepsis, but this view has been challenged [5]. CLP and CLI do not completely mimic the representative course of human perforated intestine and are difficult to control in terms of severity and predictability. The colon ascendens stent peritonitis (CASP) model, first described in mice [6] and later in rats [7], overcomes this limitation.

In this manuscript, we describe the adaptations made to improve the CASP model in terms of monitoring hemodynamics and facilitating frequent blood sampling as well as non-surgical treatment.

## Main text

### Materials and methods

These experiments were performed as a pilot of a project aiming to explore the effects of a new endotoxin scavenger as a randomized placebo-controlled trial, the results of the main experiments are not published in this manuscript.

The experiment was reviewed and approved by the Animal Care and Experimentation Committee of the Canton of Bern, Switzerland (26169 BE6/15) and followed the Swiss national guidelines for the performance of animal experiments. Male Wistar rats aged 8–9 weeks, weighing 380 g in average were obtained from Janvier Labs (Le Genest‐Saint‐Isle, France) and kept in individually ventilated cages (IVC) with controlled 12 h light/dark cycles at 22 ± 2 °C at the Central Animal Facility of the University of Bern. Food and water were provided ad libitum. A total of 56 animals were included (4 groups: 20 animals sham surgery ± scavenger/placebo, 36 animals CASP ± scavenger/placebo).

### Anesthesia

Anesthesia was induced with 6% sevoflurane (Sevorane, AbbVie, Baar, Switzerland) in oxygen in an induction chamber, and 50 µg/kg fentanyl (Janssen-Cilag, Zug, Switzerland) was added intraperitoneally. The animals were intubated using a modified 2.0 mm Angiocath (BD, Allschwil; Switzerland) and mechanically ventilated with a small animal ventilator (KTR-4 Rodent Ventilator, Hugo Sachs, March, Germany). Anesthesia was maintained with sevoflurane 2.5–3.5%, and analgesia with additional 50 µg/kg fentanyl administered subcutaneously 30 min later. The animal was placed on an operation table with an in-built feedback controlled heater system (TCAT-2, Harvard Apparatus, Hugo Sachs, March, Germany) aiming for a constant body temperature of 37 °C.

### Preparation

After incision of the right groin, we tunneled two polyurethan catheters (PU 40, SAI Infusion Technologies, Lake Villa, IL, USA with a redon needle/wound drainage trocar from the neck to the groin.The catheters were then equipped with a 1.5 to 2 cm tip made of PE-50, and filled with heparinized sterile normal saline solution (5000 I.U. heparin in 1000 ml NaCl 0.9%) via a blunt needle and luer lock syringe on the cranial part. The femoral artery and vein were microsurgically exposed, cannulated and secured with a suture. Before skin closure, the catheters were additionally bend as a loop and secured twice with sutures. The animals were turned in prone position and the catheters secured with a subcutaneous loop and suture at the neck. We channeled the catheters trough a tethered harness (SAI Infusion Technologies, Lake Villa, IL, USA) and connected them via a two channel swivel with 2 purge systems (syringe drivers) to guarantee free movement of the animal and integrity of the catheters. After drawing a first arterial and venous blood sample, both purge lines were continuously flushed with 1.5 ml/h Glucose 5% in H_2_O 2:1 and heparin (5000 I.U. heparin in 1000 ml GS), to the arterial line purge we added buprenorphine (Temgesic, Reckitt Benckiser Switzerland, Wallisellen) to achieve a concentration of 1 µg/ml. The animals were weaned from the ventilator, extubated and placed for recovery from surgery in a cage equipped with a swivel mount. Duration of surgery was 50–60 min.

### Colon ascendens stent peritonitis

6 hours after finishing the preparation the animals were re-anesthetized with sevoflurane 6% in oxygen, supplemented with 20 μg/kg buprenorphine subcutaneously. Anesthesia was maintained with 2.5 to 3.5% sevoflurane in oxygen via facemask. A median laparotomy was performed, and the coecum and the ascending colon identified and exposed. A small incision was made about 1 cm distal the iliocoecal valve at the anti-mesenterial side, and a 12 French PU catheter of 1.5 cm length was inserted. This stent was additionally fixed to the colon by a suture. Patency was tested by instillation of saline into the stent, with spontaneous emptying of feces afterwards, or gentle squeezing the coecum until intestinal content was visible in the opening of the stent. The animals in the sham group received the same surgery, omitting colostomy, with the stent only sutured at the outer side of the colon ascendens. The intestine was packed back into the abdominal cave and the abdominal wall was closed in two layers. Surgical time was below 20 min. The animal was placed back into its cage equipped with the swivel mount. Volume replacement was started, the buprenorphine dosing was resumed. The animals were provided with water and nourishment ad libitum.

### Surgical repair and treatment

16 hours after peritonitis induction and randomization, animals were again re-anesthetized as described before. The abdominal sutures were re-opened, the stent identified, which could necessitate exploration with sterile cotton swabs to remove fibrin and detritus. Cultures from the abundant purulent ascites and detritus were taken at this time point for the main experiment. Once the stent was identified, the additional suture was cut, the stent removed and the intestinal wall closed with simple interrupted stitchings. The abdominal cavity was then rinsed with warm Ringer’s solution, and the abdominal wall closed with sutures. Surgical time was below 20 min.

Then the rats were placed in their cages with the swivel mount. All animals received an intravenous infusion of meropenem (Meronem, Pfizer Switzerland, Zurich, 75 mg per kg BW, diluted to 5 mg/ml, in 15 min) followed by a continuous infusion via the central venous line (225 mg/kg BW/24 h). Analgesia was provided continuously with buprenorphine administered via the intraarterial purge line.

Blood pressure was measured continuously with a standard transducer system linked to a BIOPAC MP100 analog–digital-converter and was acquired and analyzed with the Biopac AcqKnowledge Data Acquisition Software (BIOPAC, Goleta, CA 93117, USA). Heart rate was calculated with the blood pressure tracing.

Blood samples were drawn 16 and 40 h post peritonitis induction. After the last blood sampling the animals were euthanized by intravenous infusion of pentobarbital (150 mg/kg BW, Esconarkon, Streuli Pharma AG, Uznach, Switzerland).

## Results

All but 2 animals survived the initial surgery. In the CASP group, 4 animals (out of 36) died or were euthanized according to the score sheet after sepsis induction, before randomization. No further premature death occurred after randomization and start of the treatment. Blood sampling was achieved before peritonitis induction and before start of meropenem (6 h) in all animals. Line patency decreased over time, after 24 h, blood samples could not be obtained in 9 animals, but did not decrease further.

In the 6 animals where microbiological samples from the abdominal cavity were drawn at repair, polymicrobial growth of gram negative and gram positive bacteria in great quantity could be documented as a proof of the infection with intestinal pathogenes.

**Fig. 1:**
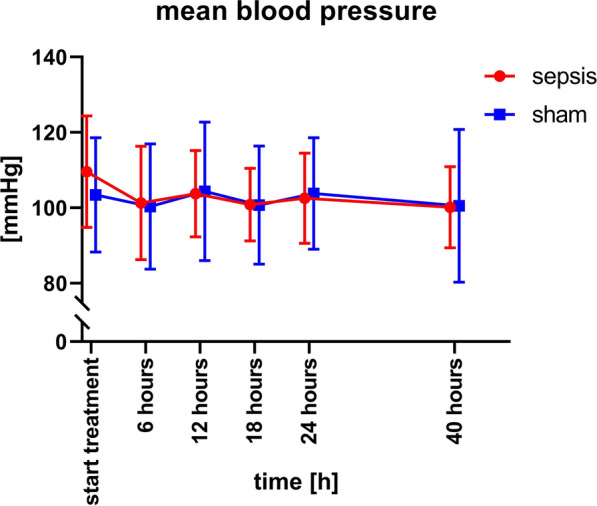
Blood pressure. Time course of blood pressure und septic (red) and sham (blue) animals. Note that measurement of blood pressure began 16 h after sepsis induction /sham surgery, when treatment with surgery and meropenem started. There are no significant differences between the groups

**Fig. 2:**
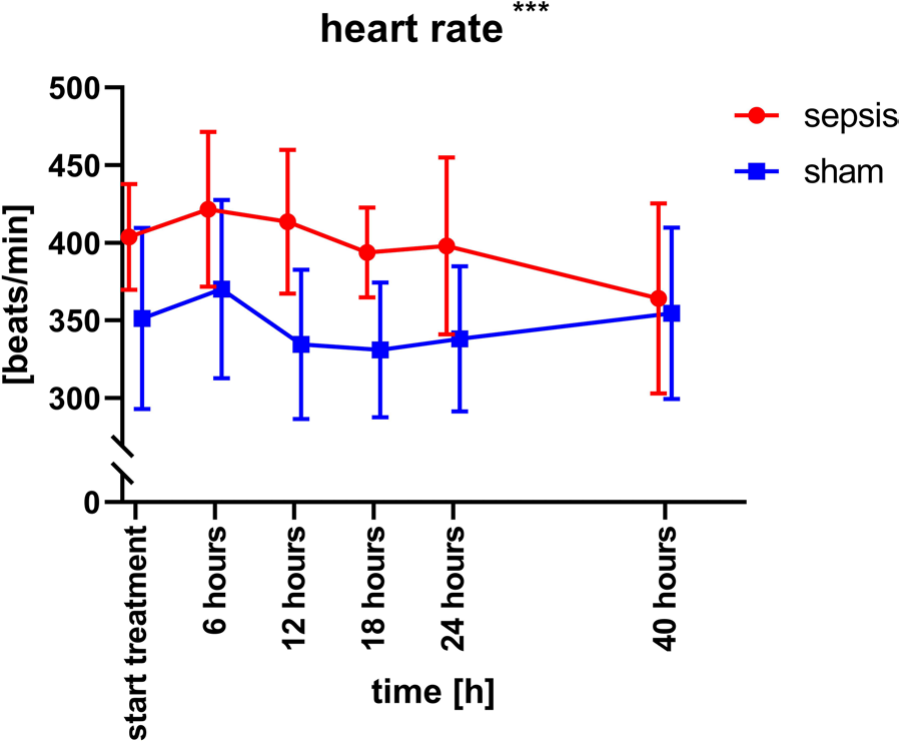
Heart rate. Time course of heart rate und septic (red) and sham (blue) animals. Note that measurement of heart rate began 16 h after sepsis induction /sham surgery, when treatment with surgery and meropenem started. The difference between the groups is significant (p = 0.005)

Because of the relatively long tubing system and connection via the swivel system, blood pressure tracings were damped, so. We relied on mean arterial pressure. Although clear signs of peritonitis were seen, and cultures grew abundant bacteria, systemic hypotension did not occur (mixed effects model for repeated measures data, p = ns, Fig. 1). Heart rate was significantly higher in the septic animals (mixed effects model, p = 0.005 for group, p = 0.02 for time, group x time factor: p = 0.04, Fig. 2).

In conclusion, the addition of the arterial and venous catheters in the CASP model expands the research possibilities of the model. With these catheters, fluid management and anti-infective as well as analgesic treatment can be individualized, and blood sampling is facilitated. By analogy with known literature, where needle size, number of punctures and length of ligation modifies severity of sepsis [8], and in view of the experience with patients, where timing of repair is crucial, we believe that this model can be used in a large variety of clinically relevant experiments.

## Limitation

Traditional models have disadvantages depending on the research questions. Lipopolysaccharide injection models do not produce sepsis, but endotoxin shock; injection of bacteria or a defined amount of bacteria or fecal slurry lack the pathophysiology of gut discontinuation. In surgical models of abdominal sepsis abscess formation frequently occurs. This does not reflect the current medical scenario, where surgical treatment with closure of the gut discontinuation and lavage is attempted. The CASP model overcomes this drawback with surgical repair of the damage. Continuous bacterial contamination renders this model moderately controllable, but adaptation of the model can be accomplished by different sizes of the stent [9], and by different timing of surgical repair. Continuous blood pressure and heart rate monitoring can guide fluid management with frequent adjustments. Together with the venous catheter, both lines enable blood sampling without posing additional stress to the animal, and safe and reliable dosing of medication.

In this pilot experiment we found that combining the vessel cannulation and the CASP surgery in one single operation leads to an unacceptable high mortality. After modification and introducing a recovery period between both procedures the mortality during surgery declined. It is quite probable that the staged surgeries in this model induce inflammatory responses on its own and interfere with the results and outcomes on each step, but we have not quantified this effect. Another problem was failure to sample blood because of blocked catheters. Although we added heparin to the purge fluids, in some animals the catheter was partially clotted or developed a valve-like phenomena. With two lines in place, we could use the second line in some instances, but in 12%, no blood sampling was possible. We recommend a tubing system with biocompatible heparin bonded surfaces, or adding an anticoagulant to the animal, which would in turn increase the risk of hemorrhage during septic shock.

Because of the long tubing and the connection with a swivel, the arterial blood pressure curve was damped. Since tissue perfusion depends on mean arterial blood pressure this is not a major drawback [10]. It could be criticized that this model did not produce relevant hypotonia and shock, as it would be expected in severe sepsis. Because of positive cultures of abundant bacteria with different species in the peritoneum, and the marked differences in heart rate between the septic and the sham animals, we are certain that sepsis and shock occurred, but treatment with intravenous fluids via purge lines and antibiotics prevented clinical deterioration and death.

A general problem in research is the use of younger animals, as we have done. Age is a well-known contributor to mortality in patients, and in animal models of sepsis [11]. Generalizability to the elderly human population may be challenging.

## Data Availability

The datasets used and/or analyzed during the current study are available from the corresponding author on reasonable request.

## Abbreviations

CLP: Cecal ligation and puncture
CLI: Cecal ligation and incision models
CASP: Colon ascendens stent peritonitis
